# Detailed analysis of the transverse arch of hallux valgus feet with and without pain using weightbearing ultrasound imaging and precise force sensors

**DOI:** 10.1371/journal.pone.0226914

**Published:** 2020-01-09

**Authors:** Hala Zeidan, Eguchi Ryo, Yusuke Suzuki, Hirotaka Iijima, Yuu Kajiwara, Keiko Harada, Kengo Nakai, Kanako Shimoura, Koji Fujimoto, Masaki Takahashi, Tomoki Aoyama

**Affiliations:** 1 Department of Physical Therapy, Kyoto University, Kyoto, Japan; 2 Department of System Design Engineering, Keio University, Yokohama, Japan; 3 Department of Physical Therapy, Kio University, Nara, Japan; 4 Human Brain Research Center, Kyoto University, Kyoto, Japan; University of California Davis, UNITED STATES

## Abstract

**Background:**

Hallux valgus is the most common forefoot deformity and affects the transverse arch structure and its force loading patterns. This study aims to clarify the differences in the transverse arch structure and the force under the metatarsal heads individually, between normal feet and hallux valgus feet, and between hallux valgus feet with pain and without pain. We further test the association between the parameters of the transverse arch and hallux valgus angle and between the parameters and pain in hallux valgus.

**Methods:**

Women’s feet (105 feet) were divided into normal group (NORM) and hallux valgus group (HVG); and further into subgroups: hallux valgus without pain (HV Pain (-)) and hallux valgus with pain (HV Pain (+)). Transverse arch height and metatarsal heads height were measured using weight-bearing ultrasound imaging. Force under the metatarsal heads was measured using force sensors attached directly on the skin surface of the metatarsal heads. The measurements were taken in three loading positions: sitting, quiet standing and 90% weight shift on the tested foot. Differences between the groups were compared using Student t-test and Wilcoxon Exact test. Multivariate logistic analysis with adjustment for physical characteristics was also conducted.

**Results:**

Transverse arch height was significantly higher in HVG than in NORM in all positions; there were no significant differences between HV Pain (+) and HV pain (-). Lateral sesamoid was significantly higher in HVG and HV Pain (+) than in NORM and HV Pain (-) respectively when bearing 90% of the body weight unilaterally. There was a trend of higher forces under the medial forefoot without significant difference. Transverse arch height and lateral sesamoid height were associated with the hallux valgus angle, while lateral sesamoid height was associated with forefoot pain in hallux valgus deformity.

**Conclusions:**

This study shows the differences in the transverse arch structure between normal feet and feet with hallux valgus, and between hallux valgus feet with and without pain. This finding is noteworthy when considering future treatments of painful feet, notably the height of the lateral sesamoid which seems to play a role in forefoot pain.

## Introduction

The foot is the only body part that is in contact with the ground [1, 2] and the forefoot is the only foot segment that is in contact during the terminal stance phase of gait [1]. Three arches help the foot perform its functions: the medial longitudinal arch, the lateral longitudinal arch, and the transverse arch, which is the least studied [3]. The transverse arch is located in the forefoot and formed by the five metatarsal heads in the frontal plane. It helps in load transmission and shock absorption to allow forward propulsion [1]. The function of the transverse arch is to center loads on the second metatarsal [3]; however, it is hypothesized that when loads are distributed unevenly, foot pathologies occur [3].

One of the most common deformities of the forefoot is hallux valgus [4–7]. It is a progressive deformity that occurs on the level of the first toe, whose alignment is altered. In this deformity, the hallux shifts laterally and the first metatarsal shifts medially [8–11] due to the weakness of the medial collateral ligament and the capsule [10]. The first ray becomes unstable and hypermobile [6, 12], the sesamoid-complex shifts [10] and the plantar flexor muscles weaken [13]. This deformity is common in the elderly [8, 14, 15], as the intrinsic muscles that stabilize the alignment of the first ray weaken with age. Several other factors also contribute to hallux valgus such as, genetics, foot anatomy and biomechanics, gender, ligament laxity, and wearing tight shoes and high heels [8, 14]. These factors can also be modifiable (body mass index and footwear) and non-modifiable (gender and foot structure) [16]. Hallux valgus is linked to foot pain, decreased quality of life, foot function and mobility, and increased risk of falling related to gait instability [6, 9, 13–16].

In its turn, foot pain as well affects daily life activities and quality of life [17], balance and gait [17, 18] and falls, and it leads to biomechanical disfunctions [18]. Foot pain is mostly located in the forefoot and toes, in women more than in men, and those with hallux valgus deformity are at higher risks [18]. Factors affecting foot pain are local (structural) and systematic (hallux valgus, dermatological problems, osteoarthritis, …) [18].

Foot pain mechanism is not yet fully understood and past studies have called for detailed and accurate examination of the biomechanics of the foot [17, 18]. Pain in hallux valgus can be caused by local mechanical stimuli (weakness of the plantar flexor muscles) [1, 8, 13, 19], dynamic structure of the foot and the ankle as well as other factors such as poor health, high occupational physical activity level and lifestyle [8]. Pain also affects gait [9] and we think that it should hence be considered when giving treatments or assigning insoles for the elderly–who are at high risk of falls [17, 20, 21]. Pain in the metatarsals is also linked to changes in the transverse arch [3]. The transverse arch of individuals with hallux valgus can be changed as hallux valgus deformity distorts the normal alignment of the hallux and the first metatarsal [8–11] and affects the structure of the transverse arch (such as transverse arch height (TAH)) [22], and the force loading patterns in the forefoot [10, 15, 23, 24].

During the terminal stance phase of normal gait, loads are received and transferred to the hallux and the first metatarsal head (1MTH) [10, 25]. The subluxation in hallux valgus may interfere with the proper force propulsions and alter the pressure in the forefoot [10, 15, 23, 24] as the ability of the hallux and 1MTH to bear weight is reduced [10], thereby altering the loading patterns [6, 10]. Force loading patterns also differ on the weight-bearing loads. Previous reports on loading patterns under the forefoot in individuals with hallux valgus are inconsistent and controversial [13–15, 26] with some studies reporting greater loads in hallux valgus individuals than in controls [27]; the contrary [28]; as well as no differences between the two groups [29]. Other studies have also found higher loads on the medial forefoot and lower loads on the lateral forefoot [4]; lesser loads on the 1MTH and higher loads on lesser toes or second metatarsal head (2MTH) and third metatarsal head (3MTH) [1, 12]; higher loads on 1MTH, 2MTH and 3MTH and lesser loads on forth metatarsal head (4MTH) and fifth metatarsal head (5MTH) [9]; higher loads on the hallux and lesser toes and lateral metatarsals [15]; and lesser loads on the medial forefoot [9]. Loading patterns in hallux valgus can also be affected by pain as the individuals may acquire an adaptive foot posture to avoid loads on the painful areas [6, 14]. To our knowledge, previous studies on the forefoot have divided it into sections (such as one section for the 1MTH, another combining the 2MTH, 3MTH and 4MTH together, and a third section for the 5MTH [1, 30], or a section for the 1MTH, another for the 2MTH and a third one for the lateral metatarsal heads [31, 32]) instead of individual measure of the metatarsal heads and a few studies have compared hallux valgus with and without pain [9, 24, 33]. No distinction between the individual force under the metatarsal heads and between feet with pain or without pain could affect the results of the study. By measuring the loading pattern, it is possible to estimate the effect that it has on functional and structural deformities [14].

The transverse arch is the least studied arch of the foot and detailed data about it are lacking. Since foot structure is cited as a non-modifiable risk factor for hallux valgus [16] and that it also affects foot pain [18], it is important to understand the differences in the structure of the transverse arch and the differences in loading patterns between normal feet and hallux valgus feet, and also between hallux valgus feet with and without pain. Therefore, this study contains two groups (comparing normal feet and hallux valgus feet) and two subgroups (comparing hallux valgus feet with pain and hallux valgus feet without pain). We chose to measure the structure and the force in three different loading positions (sitting, quiet standing and 90% weight shift) to see how loading affects the structure and force of the transverse arch. Thus, this study’s main aim is to clarify the characteristics of hallux valgus feet compared to normal feet; and hallux valgus feet with pain compared to hallux valgus feet without pain. This study’s secondary aim is to find which variables (TAH, sesamoid rotation angle (SRA), metatarsal heads’ height, force under the metatarsal heads) are associated with either hallux valgus angle (HVA) or with pain. We hypothesize that hallux valgus feet will have higher TAH and SRA, and higher forces under the 1MTH compared to normal feet, and that these parameters will be associated with HVA. We also hypothesize the same for hallux valgus feet with pain compared to those without pain: a higher TAH would lead to improper transverse arch function, such as improper shock absorption (as example: caused by the rigidity of the arch) [22], leading to pain in the forefoot (as example: caused by repetitive stress without spring effect); and that these parameters will be associated with foot pain.

## Methods

Female participants were recruited upon their request for participation during a healthcare event for the elderly which was advertised in public information magazines and occurred in Kyoto University, in August and September 2017. The approval number of this study is R0450-1 and it is in accordance with the Declaration of Helsinki and approved by the Kyoto University Graduate School and Faculty of Medicine. Explanation about the study and the measurements were done and written consents were obtained before the measurements.

Exclusion criteria were past surgeries in the lower limbs, injuries in the lower limbs during the last year, dependence, and inability to complete the test-positions alone. The total number of women was 68 women reduced to 63 women after applying the exclusion criteria.

Furthermore, the participants were asked if they had pain in the forefoot (both right and left feet) by answering a ‘‘Yes/No” question. The questionnaire was self-reported and did not ask about the intensity of the pain nor about the specific location of the pain. There were 126 feet in total, reduced to 105 feet after excluding the feet with pain but without hallux valgus deformity. Hallux valgus deformity was identified by measuring the HVA using a goniometer [34, 35]. Feet with a HVA lesser than 20 degrees were consider normal feet and feet with a HVA equal or higher than 20 degrees were considered as feet with hallux valgus deformity. This method was chosen in accordance to the Japanese Orthopedic Society criteria, where severity of hallux valgus is classified as mild at an angle between 20 and 30 degrees, moderate at an angle between 30 and 40 degrees and severe at an angle higher than 40 degrees [34]. Each foot was considered as an individual sample and divided into groups. In the first step analysis, the 105 feet were divided into two groups: 1) normal feet group (NORM, n = 71) and 2) hallux valgus feet group (HVG, n = 34). In the second step of the analysis, on the hallux valgus feet were divided into two subgroups: 1) hallux valgus without pain (HV Pain (-), n = 18) and 2) hallux valgus with pain (HV Pain (+), n = 16), depending on the self-reported questionnaire about forefoot pain.

### Hallux valgus angle

HVA was measured barefooted in standing position with a finger goniometer. One hand of the goniometer was placed on the medial aspect of the hallux and the other hand was placed on the medial aspect of the first metatarsal. This method was described and shown reliable by test-retest in another previous publication [35].

### Weight-bearing plantar ultrasound imaging device

**Fig 1:**
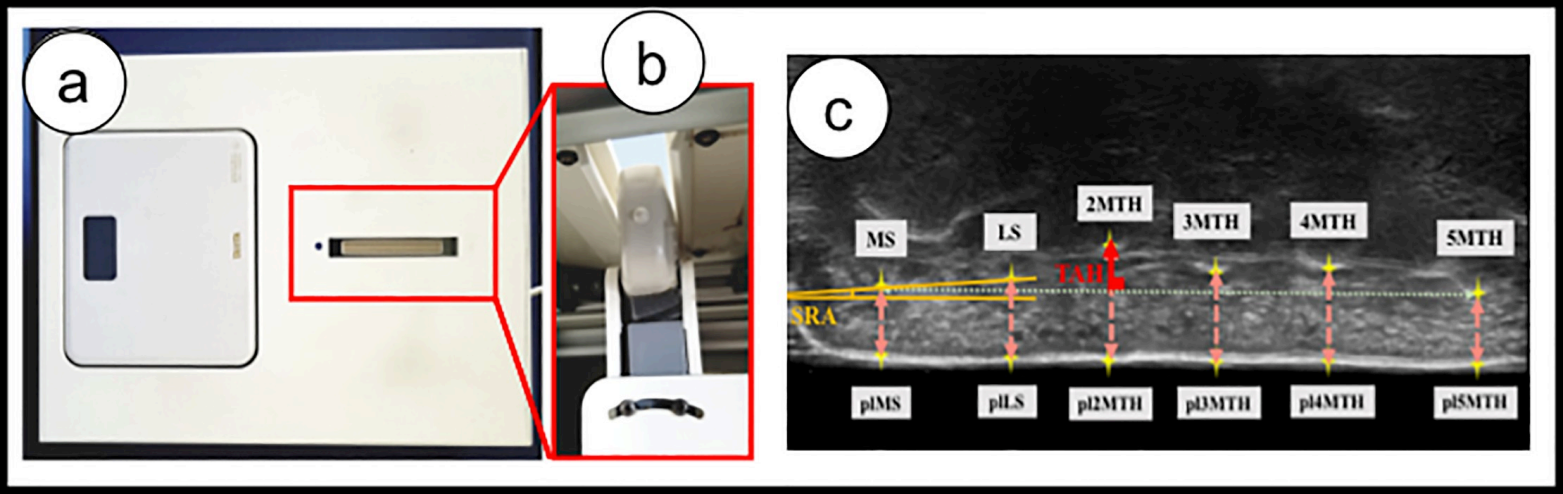
Weight-bearing plantar ultrasound imaging device and ultrasound images. (a) WPUID constructed with an internal probe allowing coronal views of the transverse arch; (b) ultrasound probe inserted upside-down and an upper opening for a gel pad to be placed on the probe; (c) shows the ultrasound image: the lowest point of the MS, LS, 2MTH, 3MTH, 4MTH and 5MTH as well as their plantar projections were marked by yellow stars. TAH (red line) is the distance from 2MTH perpendicular to the line passing through MS and 5MTH. Metatarsal heads’ height (orange dotted lines) is the distance between the lowest point of the bone and its plantar surface marker. SRA (yellow angle) is the angle between the line passing through MS and LS and the line passing through MS and 5MTH. US: ultrasound; WPUID: Weightbearing Plantar Ultrasound Imaging Device; MTH: metatarsal head; TAH: transverse arch height; SRA: sesamoid rotation angle; MS: medial sesamoid; LS lateral sesamoid; 2MTH: second metatarsal head; 3MTH: third metatarsal head; 4MTH: forth metatarsal head; 5MTH: fifth metatarsal head; pl: plantar surface.

A weight-bearing plantar ultrasound imaging device (WPUID) was constructed with an internal probe allowing coronal views of the transverse arch. The WPUID is shaped as a rectangular empty box and has an opening allowing for an ultrasound probe (Noblus, Hitachi Aloka Medical, Tokyo, Japan) (Fig 1) to be inserted upside-down and a weight scale (Fig 1). This opening also allows gel pads to be placed above the upside-down probe to test the foot in weightbearing and to take ultrasound images. The weight scale allows for control of weight shifts during measurements. Ultrasound images were taken using B-mode with a frequency of 9.0 MHz after confirming the lowest points of the epiphysis of the medial sesamoid (MS), the lateral sesamoid (LS), 2MTH, 3MTH, 4MTH and 5MTH. These ultrasound images were previously shown to be in agreement with computerized tomography ultrasonograms [36] and this method was previously used to view the structure of the transverse arch [22, 36]. One image was taken in each of the three measurement positions (detailed explanation of these positions follows in the text) resulting in three ultrasound images per foot.

### Analysis of the ultrasound images taken with the weight-bearing plantar ultrasound imaging device

Ultrasound images were analyzed using ImageJ software (National Institute of Health, Washington, DC, USA), after transferring to a computer. Fig 1 represents an ultrasound image and the measured parameters. The lowest points of the MS, LS, 2MTH, 3MTH, 4MTH and 5MTH were marked by a yellow star. The projection of these points on the plantar surface was also marked; resulting in a total of 12 markers in one ultrasound image. From these markers, TAH, SRA and the height of each metatarsal head were calculated using Excel (Microsoft, Redmond, WA) as follows. The TAH is the distance between 2MTH and the line passing through MS and 5MTH as calculated by the following formula which was previously measured by Nakayama et al. [37]: L2M/LM5*100 (L2M is the line passing by 2MTH and L2M, and LM5 is the line passing by MS and 5MTH). The height of the metatarsal heads was measured between the marker of the lowest point of the metatarsal head and the plantar marker of the same metatarsal head. SRA was measured as the angle between the line passing through MS and LS and the line passing through MS and 5MTH.

### Force sensing device using force sensors

**Fig 2:**
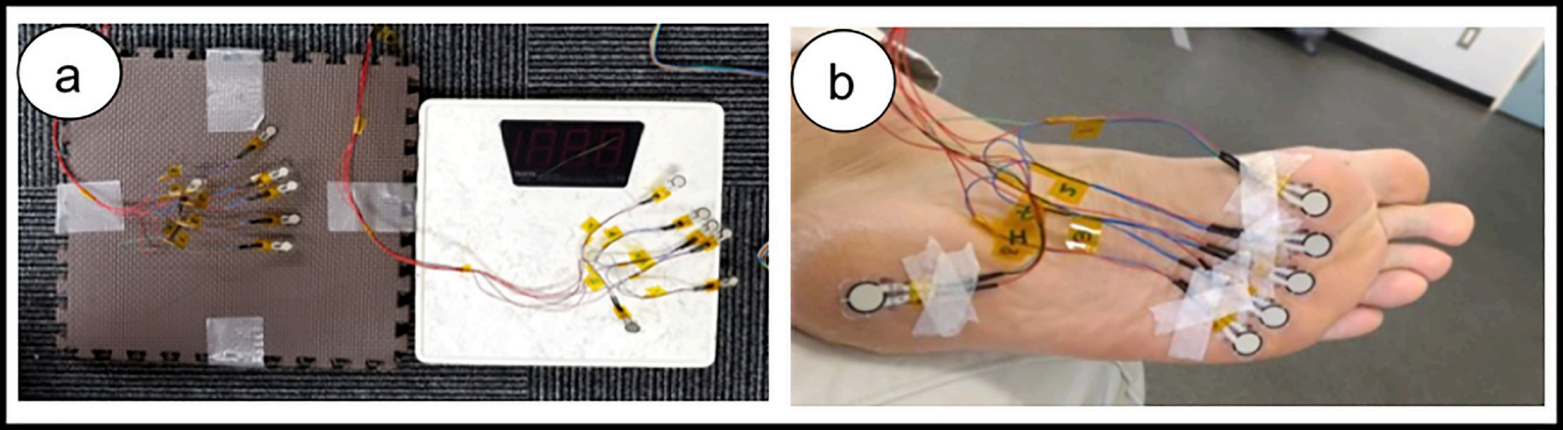
Force sensing device using force sensors. (a) Force sensors’ system with the 12 sensors and a weight scale; (b) shows the sensors stuck on the skin surface of the metatarsal heads after palpation.

Six force sensors (FlexiForce Standard Model A301, Tekscan, South Boston, US.) were attached on the plantar surface of each measured foot. The sensing area is a 9.53-mm diameter circle at the end of the force sensor. The resistance of the sensing element changes in an inversely proportional relationship to the applied force. Each sensor was calibrated using Press Force Sensor 9313AA2 (Kistler, Winterthur, CH) before the first use. A circuit board containing a microcontroller and AD converters was developed. A ribbon cable was used to connect the force sensors to the circuit boards. The sensors were connected to a laptop computer using custom software (C++), and data were collected through a USB connection. In total, 12 sensors were connected (Fig 2). The MTHs were palpated on the plantar surface of the foot and the convenient sensor was attached to the skin. Six sensors, five on the metatarsal heads and one on the heel, were attached on both feet (Fig 2). Measurements were taken from both feet in each of the three positions described above. The force values were normalized by dividing each value by the total value of forces of the foot in order to standardize the data and compare it equally.

### Measurement positions

Measurements on both devices were taken in three positions as follows. These positions were used in a previous study [22].

**Fig 3:**
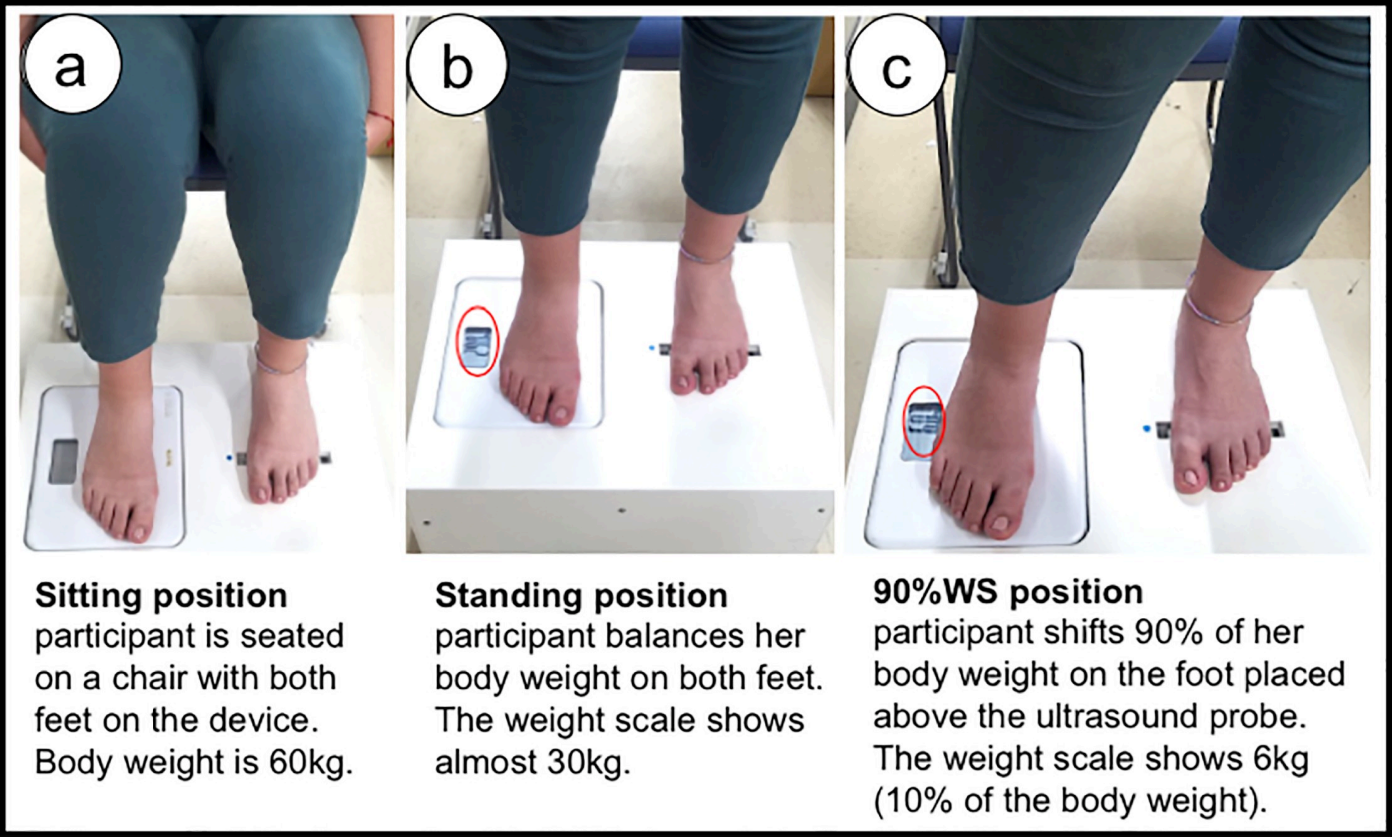
Measurement positions. (a) sitting position: participant is seated on a chair with knees 90 degrees flexed, both feet placed for measurement; (b) standing position: participant keeps her feet as placed in the former position and stands up. The weight scale is used to balance half the weight on both feet; (c) 90%WS position: 90% of body weight is placed on the tested foot while 10% of the body weight is monitored on the weight scale under the nontested foot. 90%WS: 90% weight shift.

- **Sitting position:** the participant was seated with both feet touching the surfaces of the measurement devices (Fig 3).
- **Standing position:** the participant was standing with feet shoulder width apart and weight was distributed equally on both feet. A scale was used to monitor the weight distribution (Fig 3).
- **90% Weight Shift position (90%WS):** the participant was shifting 90% of her body weight on the tested foot and 10% on the non-tested foot and a scale was used to monitor this shift. Once the body weight shifts were maintained in this position, we asked the participant to slightly shift her weight on the forefoot of the tested foot (Fig 3). This was done to simulate the terminal stance phase of gait where 90 to 110% of body weight is being transferred unilaterally to the forefoot and hallux [25].

### Statistical analysis

The sample size was decided based on other studies about hallux valgus or ultrasound devices assessing the foot or plantar pressure studies [38–40]. Statistical analysis was performed using JMP Pro 14 software (SAS Institute, Cary, NC, USA) and statistical significance was set at p < 0.05. First, Shapiro-Wilk test was used to check the normality of the data. Student’s t-test was used to compare TAH, SRA and metatarsal heads’ heights between NORM and HVG groups. The same test was used to compare these parameters between HV Pain (-) and HV Pain (+) groups. This test was used because these results were normally distributed. Wilcoxon Exact test was used to compare the force under the metatarsal heads between NORM and HVG groups. The same test was used to compare these parameters between HV Pain (-) and HV Pain (+) groups. This test was used because the force parameters were not normally distributed. Afterwards, the results that were significantly different were subjected to a multivariate logistic analysis (as our independent variables, HVA or pain, were binomial variables) [41], with HVA or pain as independent variables and the structure of the transverse arch and force under the metatarsal heads outcomes as dependent variables, with adjustment for physical characteristics that were significantly different between the groups in order to avoid bias, to demonstrate any associations of the structure of the transverse arch and force under the metatarsal heads with HVA or the structure of the transverse arch and force under the metatarsal heads with pain.

## Results

The physical characteristics of NORM and HVG groups are presented in Table 1, and those of HV Pain (-) and HV Pain (+) groups in Table 2.

**Table 1:** Physical characteristics of NORM and HVG groups. The results are represented as mean ± SD, compared using Student’s t-test. P-value was set as 0.05.

	NORM	HVG	p-Value
**Age (years)**	58.56 ± 6.48	61.88 ± 7.69	0.0340*
**Body Height (cm)**	156.58 ± 4.78	157.49 ± 4.74	0.3605
**Body Weight (kg)**	59.04 ± 12.38	55.93 ± 8.23	0.1305
**BMI (kg/m**^**2**^**)**	24.07 ± 4.89	22.49 ± 2.64	0.0345*

NORN normal feet group; HVG hallux valgus feet group; BMI body mass index.

* significant p-Value.

**Table 2:** Physical characteristics of HV Pain (-) and HV Pain (+) groups. The results are represented as mean ± SD, compared using Student’s t-test. P-value was set as 0.05.

	HV Pain (-)	HV Pain (+)	p-Value
**Age (years)**	61.22 ± 8.57	62.63 ± 6.78	0.60
**Body Height (cm)**	158.5 ± 5.72	156.35 ± 3.11	0.18
**Body Weight (kg)**	58.87 ± 8.72	52.63 ± 1.60	0.0227*
**BMI (kg/m**^**2**^**)**	23.35 ± 2.50	21.52 ± 2.53	0.0425*

HV Pain (-) hallux valgus feet without pain group; HV Pain (+) hallux valgus feet with pain group; BMI body mass index.

* significant p-Value.

The participants of NORM and HVG groups had a significantly different age range (*p* = 0.0340), similar body heights (*p* = 0.3605), similar body weights (*p* = 0.1305), a significantly different body mass index (BMI) (*p* = 0.0345) and a significantly different HVA (*p* < 0.0001).

The participants in HV Pain (+) and HV Pain (-) groups had similar ages (*p* = 0.60), similar body heights (*p* = 0.18), significantly different body weight (*p* = 0.0227), a significantly different BMI (*p* = 0.0425) and no significant difference in HVA (*p* = 0.3840).

The results of TAH, SRA and metatarsal heads’ height of NORM and HVG groups are presented in Table 3, and those of HV Pain (-) and HV Pain (+) groups in Table 4.

TAH was significantly higher in HVG compared to the NORM in all positions (Sitting: *p* = 0.0125 / Standing: *p* = 0.0081 / 90%WS: *p* = 0.0441). In sitting, SRA was significantly higher in HVG compared to NORM while in standing and in 90%WS, there was no difference between the groups (Sitting: *p* = 0.0267/ Standing: *p* = 0.445 / 90%WS: *p* = 0.8016). MS height was significantly lower in HVG compared to NORM in all positions (*Sitting*: *p* = 0.0013 / Standing: *p* = < .0001 / 90%WS: *p* = 0.0314). LS height was significantly higher in HVG in 90%WS (*p* = 0.0209), whereas 5MTH height was significantly lower in HVG in standing and 90%WS (Standing: *p* = 0.0026 / 90%WS: *p* = 0.0429). The heights of the other metatarsal heads were not significantly different in all positions. These results are shown in Table 3.

**Table 3:** TAH, SRA and metatarsal heads’ heights between NORM and HVG in sitting, standing and 90%WS positions; results presented as mean ± SD, compared using Student’s t-test.

	Sitting			Standing			90%WS		
	NORM	HVG	p-Value	NORM	HVG	p-Value	NORM	HVG	p-Value
**TAH (mm)**	4.96 ± 2.08	6.02 ± 1.93	0.0125*	4.82 ± 2.07	5.90 ± 1.82	0.0081*	4.77 ± 1.95	5.46 ± 1.43	0.0441*
**SRA (degree)**	4.97 ± 2.49	6.22 ± 2.70	0.0267*	5.45 ± 2.76	5.92 ± 3.02	0.445	5.94 ± 3.19	5.78 ± 2.74	0.8016
**MS Height (mm)**	6.16 ± 1.15	5.46 ± 0.91	0.0013*	6.06 ± 1.01	5.22 ± 0.85	< .0001*	5.99 ± 0.90	5.61 ± 0.81	0.0314*
**LS Height** **(mm)**	8.42 ± 1.45	8.84 ± 1.35	0.1419	8.62 ± 1.53	8.78 ± 1.49	0.6162	8.73 ± 1.47	9.46 ± 1.49	0.0209*
**2MTH Height (mm)**	10.92 ± 1.98	11.49 ± 1.82	0.1461	10.62 ± 1.98	11.06 ± 1.81	0.2577	10.56 ± 1.91	10.83 ± 1.79	0.4875
**3MTH Height (mm)**	9.75 ± 1.57	9.66 ± 1.81	0.8195	9.40 ± 1.62	9.11 ± 1.69	0.4164	9.40 ± 1.64	9.25 ± 1.52	0.6324
**4MTH Height (mm)**	8.86 ± 1.30	8.52 ± 1.54	0.2644	8.54 ± 1.48	8.06 ± 1.44	0.1131	8.64 ± 1.58	8.32 ± 1.54	0.3267
**5MTH Height (mm)**	7.19 ± 1.02	6.94 ± 1.08	0.2591	6.76 ± 0.99	6.12 ± 0.99	0.0026*	6.64 ± 1.07	6.14 ± 1.18	0.0429*

NORM normal feet group, HVG hallux valgus group, MS medial sesamoid, LS lateral sesamoid, MTH Metatarsal Head.

* significant p-Value

**Table 4:** TAH, SRA and metatarsal heads’ heights between HV Pain (-) and HV Pain (+) in the sitting, standing and 90%WS positions; results presented as mean ± SD, compared using Student’s t-test.

	Sitting			Standing			90%WS		
	HV Pain (-)	HV Pain (+)	p-Value	HV Pain (-)	HV Pain (+)	p-Value	HV Pain (-)	HV Pain (+)	p-Value
**TAH (mm)**	5.81 ± 1.91	6.25 ± 2.0	0.5108	5.61 ± 1.79	6.22 ± 1.86	0.3351	5.22 ± 1.50	5.72 ± 1.34	0.313
**SRA (degree)**	6.25±3.13	6.18 ± 2.22	0.9447	5.56 ± 3.16	6.32 ± 2.90	0.4726	5.62 ± 2.98	5.97 ± 2.53	0.7105
**MS Height (mm)**	5.51 ± 0.90	5.41 ± 0.95	0.7369	5.34 ± 0.81	5.07 ± 0.89	0.3651	5.58 ± 0.88	5.63 ± 0.76	0.8608
**LS Height (mm)**	8.48 ± 1.42	9.25 ± 1.81	0.0934	8.87 ± 1.50	8.67 ± 1.52	0.7073	8.89 ± 1.50	10.10 ± 1.21	0.0144*
**2MTH Height (mm)**	11.02 ± 1.80	12.02 ± 1.74	0.1099	10.60 ± 1.88	11.59 ± 1.62	0.1055	10.30 ± 1.97	11.42 ± 1.40	0.0647
**3MTH Height (mm)**	9.27 ± 2.02	10.11 ± 1.50	0.1728	8.99 ± 1.90	9.25 ± 1.46	0.6492	8.93 ± 1.70	9.60 ± 1.23	0.195
**4MTH Height (mm)**	8.26 ± 1.78	8.81 ± 1.21	0.3034	8.11 ± 1.70	8.0 ± 1.15	0.8278	8.06 ± 1.63	8.62 ± 1.42	0.2915
**5MTH Height (mm)**	6.67 ± 1.25	7.24 ± 0.78	0.1225	5.94 ± 1.16	6.32 ± 0.73	0.2636	5.89 ± 1.11	6.43 ± 1.23	0.1884

HV Pain (-) hallux valgus feet without pain group, HV Pain (+) hallux valgus feet with pain, MS medial sesamoid, LS lateral sesamoid, MTH Metatarsal Head.

* significant p-Value.

Meanwhile, TAH tended to be higher without significance in HV Pain (+) group compared to HV Pain (-) group in all positions (Sitting: *p* = 0.5108 / Standing: *p* = 0.3351 / 90%WS: *p* = 0.313). SRA was not different in both groups; but it tended to be higher in HV Pain (+) group compared to HV Pain (-) group in standing and in 90%WS (Sitting: *p* = 0.9447 / Standing: *p* = 0.4726 / 90%WS: *p* = 0.7105). The heights of the metatarsal heads showed no significant differences between HV Pain (+) and HV Pain (-) groups in all positions. Only LS in 90%WS showed increased height with significance in HV Pain (+) compared to HV Pain (-) (*p* = 0.0144). These results are shown in Table 4.

The results of force under the metatarsal heads of NORM and HVG groups are presented in Table 5, and those of HV Pain (-) and HV Pain (+) groups in Table 6.

**Table 5:** Force under the metatarsal heads between NORM and HVG in sitting, standing and 90%WS positions; results presented as mean ± SD, compared using Wilcoxon test.

	Sitting			Standing			90%WS		
	NORM	HVG	p-Value	NORM	HVG	p-Value	NORM	HVG	p-Value
**1MTH Force (N)**	0.10±0.09	0.11±0.11	0.3401	0.10±0.07	0.11±0.09	0.2893	0.15±0.10	0.17±0.12	0.3705
**2MTH Force (N)**	0.11±0.06	0.10±0.07	0.064	0.12±0.08	0.11±0.08	0.2056	0.20±0.09	0.21±0.13	0.3034
**3MTH Force (N)**	0.16±0.10	0.17±0.10	0.3705	0.18±0.06	0.19±0.10	0.3526	0.25±0.08	0.25±0.09	0.4018
**4MTH Force (N)**	0.13±0.07	0.11±0.05	0.0350*	0.12±0.05	0.09±0.05	0.0255*	0.16±0.06	0.14±0.05	0.1075
**5MTH Force (N)**	0.17±0.10	0.14±0.07	0.064	0.11±0.07	0.10±0.07	0.1903	0.19±0.11	0.18±0.10	0.4742
**Heel** **Force (N)**	0.33±0.16	0.37±0.16	0.0853	0.37±0.13	0.40±0.18	0.313	0.05±0.04	0.05±0.04	0.3401

NORM normal feet group, HVG hallux valgus group, MTH Metatarsal Head.

* significant p-Value

**Table 6:** Force under the metatarsal heads between HV Pain (-) and HV Pain (+) in sitting, standing and 90%WS positions; results presented as mean ± SD, compared using Wilcoxon test.

	Sitting			Standing			90%WS		
	HV Pain (-)	HV Pain (+)	p-Value	HV Pain (-)	HV Pain (+)	p-Value	HV Pain (-)	HV Pain (+)	p-Value
**1MTH Force (N)**	0.13 ± 0.14	0.10 ± 0.06	0.4391	0.09 ± 0.08	0.13 ± 0.10	0.1119	0.13 ± 0.08	0.21 ± 0.14	0.0721
**2MTH Force (N)**	0.09 ± 0.08	0.11 ± 0.06	0.2641	0.11 ± 0.08	0.12 ± 0.09	0.3603	0.20 ± 0.10	0.23 ± 0.16	0.2641
**3MTH Force (N)**	0.17 ± 0.09	0.18 ± 0.11	0.4257	0.20 ± 0.10	0.18 ± 0.11	0.2641	0.26 ± 0.08	0.24 ± 0.10	0.2869
**4MTH Force (N)**	0.10 ± 0.04	0.12 ± 0.05	0.1119	0.10 ± 0.04	0.09 ± 0.05	0.1566	0.14 ± 0.04	0.14 ± 0.07	0.3861
**5MTH Force (N)**	0.14 ± 0.07	0.13 ± 0.06	0.2869	0.13 ± 0.07	0.07 ± 0.05	0.0032*	0.21 ± 0.10	0.14 ± 0.09	0.0212*
**Heel Force (N)**	0.38 ± 0.17	0.37 ± 0.15	0.4391	0.37 ± 0.15	0.42 ± 0.21	0.2641	0.05 ± 0.04	0.05 ± 0.04	0.4257

HV Pain (-) hallux valgus feet without pain group, HV Pain (+) hallux valgus feet with pain, MTH Metatarsal Head.

* significant p-Value

There was no significant change in the force under the metatarsal heads between NORM and HVG groups in all positions, except a significantly decreased force under 4MTH in sitting and standing positions in HVG compared to NORM (Sitting: *p* = 0.0350 / Standing: *p* = 0.0255), shown in Table 5. We noticed that in 90%WS, there was a trend of higher forces under the 1MTH and 2MTH in the HVG than in the NORM, without statistical significance.

There were no significant differences in the force under the metatarsal heads between the HV Pain (+) group and HV Pain (-) group, in all positions, except a significantly decreased force under the 5MTH in HV Pain (+) in standing and 90%WS positions (Standing: *p* = 0.0032 / 90%WS: *p* = 0.0212), shown in Table 6. We again noticed that in 90%WS, there was a trend of higher forces under the 1MTH and 2MTH and lower forces under the 3MTH in HV Pain (+) than in HV Pain (-).

Lastly, the multiple logistic regression analysis results for NORM and HVG groups are represented in Table 7. For NORM and HVG, multiple logistic regression was conducted for the significant results of TAH in all positions, SRA in standing and in 90%WS, MS height in all positions, LS height in 90%WS, 5MTH height in standing and 90%WS and force under 4MTH in sitting and standing, to check whether these parameters were associated to HVA with adjustment to age and BMI. TAH was significantly associated with HVA in all positions (Sitting: p = 0.0455 / Standing: p = 0.0035 / 90%WS: p = 0.0130). SRA was not significantly associated with HVA in standing (p = 0.5519) and 90%WS (p = 0.2914). MS height was significantly associated with HVA in sitting (p = 0.0272), standing (p < .0001) and 90%WS (p = 0.0026). LS height was significantly associated with HVA in 90%WS (p = 0.0116). 5MTH height was not significantly associated with HVA in standing (p = 0.0512) and 90%WS (p = 0.2118). Force under 4MTH was significantly associated with HVA in sitting (p = 0.0286) but not in standing (p = 0.282).

**Table 7:** Association the significant parameters in NORM vs HVG with HVA, adjusted to age and BMI using multiple logistic regression.

		Multiple Logistic Regression		
	R2	Adjusted R2	95%CI	p
**Sitting TAH**	0.12	0.10	0.00–0.07	0.0455*
**Standing TAH**	0.08	0.06	0.02–0.10	0.0035*
**90%WS TAH**	0.07	0.04	0.01–0.08	0.0130*
**Standing SRA**	0.04	0.02	-0.04–0.07	0.5519
**90%WS SRA**	0.04	0.01	-0.09–0.03	0.2914
**Sitting MS Height**	0.11	0.08	-0.04 –-0.00	0.0272*
**Standing MS Height**	0.20	0.17	-0.06 –-0.02	< .0001*
**90%WS MS Height**	0.12	0.10	-0.03–0.00	0.0026*
**90%WS LS Height**	0.10	0.07	0.01–0.06	0.0116*
**Standing 5MTH Height**	0.09	0.06	-0.04–0.00	0.0512
**90%WS 5MTH Height**	0.05	0.02	-0.04–0.01	0.2118
**Sitting 4MTH Force**	0.05	0.02	-0.00 –-0.00	0.0286*
**Standing 4MTH Force**	0.02	-0.01	-0.00–0.00	0.282

NORM normal group, HVG hallux valgus group, HVA hallux valgus angle, CI confidence interval, TAH transverse arch height, SRA sesamoid rotation angle, 90%WS 90% weight shift position, MS medial sesamoid, LS lateral sesamoid, 5MTH fifth metatarsal head, 4MTH forth metatarsal head.

* significant p-Value

As for HV Pain (-) and HV Pain (+) groups, multiple logistic regression was conducted for the significant results of LS height in 90%WS and force under 5MTH in standing and in 90%WS, to check whether these significant parameters were associated with to pain with adjustment to BMI. LS height was significantly associated to pain in 90%WS (R2 = 0.17, Adjusted R2 = 0.12; 95% CI = -1.15 –-0.11; p = 0.0196). Force under 5MTH was significantly associated to pain in standing (R2 = 0.28, Adjusted R2 = 0.23; 95% CI = 0.00–0.05; p = 0.0273) but not in 90%WS (R2 = 0.14, Adjusted R2 = 0.08; 95% CI = -0.00–0.07; p = 0.0856).

## Discussion

In this study, we divided our sample into two groups: NORM and HVG; and into two subgroups of the HVG: HV Pain (-) and HV Pain (+) groups; and compared each set apart. We compared the structure of the transverse arch (such as TAH, SRA and metatarsal heads’ height), using weight-bearing ultrasound imaging. We also compared the forces under the metatarsal heads, using force sensors attached directly to the plantar surface of the five metatarsal heads. Both measurements were taken in three loading positions (sitting, quiet standing and 90% weight shift). Furthermore, we checked for association of HVA or pain with the structure of the transverse arch and force under the metatarsal heads. Concerning the structure of the transverse arch, our main results were significantly higher TAH in HVG than in NORM but no significant difference in TAH between HV Pain (+) and HV Pain (-) groups in all positions. We found significantly higher SRA in HVG compared to NORM in sitting and significantly lower MS height in all positions and significantly higher LS height in 90%WS positions. Meanwhile, only LS height was significantly higher in HV Pain (+) group compared to HV Pain (-) group in 90%WS. Concerning force under the metatarsal heads, we found trends of higher forces on the medial aspect of the forefoot without significant differences in all groups and positions. These results confirm our hypothesis that structure of the transverse arch is different between NORM and hallux valgus feet: such as higher TAH in HVG; however, only LS height was the significant parameter of the transverse arch structure between HV Pain (+) and HV Pain (-). There were no significant differences in force under the metatarsal heads either, but there were trends of higher forces on the medial aspect of the forefoot. Finally, TAH, MS and LS heights were associated to HVA, while only LS height was associated to forefoot pain in hallux valgus feet.

TAH was significantly higher in all positions in the HVG group than in the NORM group. In HVG group, the TAH may be affected by the displacement and rotation of the metatarso-sesamoid complex. The medial sesamoid and lateral sesamoid, which are connected to each other by an interosseous ligament, lay at the side of the flexor hallucis longus under the 1MTH and they subluxate away from the 1MTH in hallux valgus deformity [5]. The MS moves under the 1MTH and the LS moves in the space between 1MTH and 2MTH [10]. It is possible that when LS moves to the space between 1MTH and 2MTH, the space is tightened causing the 2MTH to elevate owing to the lack of space. Although our results are not significant, 2MTH height shows slightly higher values in HVG compared to NORM group.

This process could have affected TAH when it was measured using 2MTH.

The SRA was almost equal in all positions between NORM and HVG groups, except in sitting position. Although the metatarso-sesamoid complex is known to rotate in hallux valgus deformity [42, 43], our results showed similar angles in both groups in standing and 90%WS. We expected higher angles in HVG compared to NORM. This unexpected result could be because, in HVG, we combined the feet with pain and those without pain together, which could have affected the results when compared with NORM group.

Additionally, only the force under the 4MTH was significant in sitting and in standing positions in HVG group compared to NORM. However, we noticed a trend of higher forces under 1MTH and 2MTH in 90%WS in HVG compared to NORM. Although the difference was not statistically significant, these results resemble the ones from the study of Suzuki et al. [4] who found higher loads on the medial forefoot. Other studies showed greater hallux pressure in individuals with hallux valgus than in controls [25, 27]. A trend of higher forces under the medial forefoot could be due to the decreased height of the medial longitudinal arch owing to aging, as our participants were elderly women and it is known that the medial arch decreases in height with age [15, 44]. This fact was previously reported by Hagedorn et al. [15] who investigated elderly aged 66.2±10.5 years old. A lower medial arch pronates the foot [44] and we think it is the cause of higher load on the medial forefoot. This could be the reason we found no changes between NORM and HVG groups in this study, as both groups’ participants are relatively elderly women. We had hypothesized that TAH, SRA and forces under 1MTH would be higher in HVG than in NORM. Our hypothesis holds true in regard to TAH but not for SRA (although LS height significantly increased in 90%WS) nor for force under 1MTH, which only showed higher force trend. From this, we showed that the main different characteristics between NORM and HVG is the TAH no matter the load and LS height in higher loads (90%WS).

As for HV Pain (-) and HV Pain (+) groups, TAH was slightly increased, without statistical significance, in all positions in HV Pain (+). Furthermore, SRA, LS height and 2MTH height were increased, without statistical significance, in all positions in the HV Pain (+) group compared to HV Pain (-) group; whereas the LS height in the 90%WS position was significantly increased in the HV Pain (+) group. We think that the increased sesamoid rotation interferes with the position of the 2MTH in the HV Pain (+) group thereby causing the TAH to be slightly higher in that group. As mentioned in the previous paragraph, the MS moves under the 1MTH and the LS moves in the space between 1MTH and 2MTH [10] which could affect the position of the 2MTH. Sesamoid positions also differ in loading and offloading [5] and the metatarso-sesamoid complex pronates in different degrees depending on the degree of laxity of the ligament [42, 43]. The tendency of the TAH to be higher in the group with pain could be an indicator of lack in the shock absorption function of the transverse arch, which could be the cause of the pain.

As for the forces under the metatarsal heads, we found no significant differences between the groups, except for 5MTH where the force decreased significantly in standing and 90%WS positions in HV Pain (+) group compared to the HV Pain (-) group. Moreover, we noticed a trend of forces being present on the medial aspect of the forefoot, with slightly higher forces under the 1MTH and 2MTH and lesser force under the 3MTH in the standing and 90%WS positions in the HV Pain (+) group compared to HV Pain (-) group. In hallux valgus deformity, the first metatarsophalangeal joint is hypermobile [9, 14] because of the flexor muscles weakness [9] causing the first metatarsal and the hallux to be deficient in propelling forces properly. This weakness and lack of support causes the first ray to give way when receiving loads. As a result, it offloads and leads to dispersion of forces on the lesser toes [3, 9, 12, 14], accompanied by offloading of the lateral toes in some cases [12]. This is said to be an adaptive mechanism to avoid and alleviate discomfort and pain [6, 14]. However, HV Pain (+) group showed slightly higher 1MTH forces in standing and 90%WS positions, in comparison to the HV Pain (-) group. Loads are received on the hypermobile joint without being transferred to the lesser toes and there is more exposure to repetitive loads. Exposure to repetitive pressure causes injuries [45] and pain. Previously, higher plantar loads in barefoot and more weight-bearing were linked to pain [4, 8]. Further, we noticed that the force under the 1MTH was lower during sitting in the HV Pain (+) group compared to HV Pain (-) group, while there was higher force under the 1MTH in standing and 90%WS in HV Pain (+) group compared to HV Pain (-) group. This could be a factor affecting pain in hallux valgus deformity; however, we do not have the data on whether the participants had pain in non-weight-bearing as well as in weight-bearing positions. We had hypothesized that TAH, SRA and force under 1MTH would be higher in HV Pain (+). However, our results only showed statistical difference in LS height in 90%WS. The sample size in these groups was small which could have affected our results. From this, we showed that the different characteristics of hallux valgus feet with and without pain are LS height loading (90%WS). LS could be pressing on the tissues between 1MTH and 2MTH and causing the pain; although there may be other underlying factors which we did not look into. It is important to know the exact forces under the metatarsal heads because the altered loadings in hallux valgus deformity are a greater risk of not only other foot deformities, but also lower limb deformities and disorders [14].

From our results, we saw that there is a relation between the transverse arch structure and hallux valgus deformity. We estimate that this relation is due to the formation of the transverse arch by the five metatarsal heads and sesamoids, and the rotation of the sesamoids in hallux valgus which causes the LS to enter the space of 2MTH and increasing the TAH, on one hand. On the other hand, the transverse arch works on loads distribution and when this function is affected, it may cause hallux valgus deformity. It is however still unclear whether high transverse arch affects hallux valgus deformity or vice versa. Longitudinal studies may be needed to observe changes in the human foot. We believe that interventions should consider the structure of the transverse arch and the loading patterns to correct the small details. Past publications on interventions for hallux valgus depended on the severity and duration of the deformity, the level of pain, and the age of the individual [9, 46], ranging from surgery to orthotics [7] and specific exercises [47]. One study mentioned foot mobilization such as: manual mobilization focusing on flexion and caudal sliding of the metatarsophalangeal joints, tarsals, subtalar and ankle joint and exercise (hallux plantar flexion strengthening exercises, hallux abduction strengthening exercises and towel curl exercise) to increase the foot’s joints range of motion and toe grip strength and decrease pain [48]. Others mentioned toe-spread-out exercise [47, 49] and short-foot exercise [49]. While another one mentioned passive abduction with traction and active abduction exercises of the hallux combined with taping [50]. Finally, the use of electrical stimulation was mentioned to reduce pain and to potentially strengthen these muscles [49]. Also, a previous study on feet with high longitudinal arch management mentioned the use of orthotics and physiotherapy [51] or physiotherapy combined with conservative treatments [50]. In a deformity, the activity of the intrinsic muscles of the foot (the abductor hallucis muscle and the adductor hallucis muscle [47]) is imbalanced [47, 49]. This malfunction is a factor of hallux valgus development [48]. From the relation between HVA and TAH seen in our results, we would suggest the same management methods in increased TAH by targeting the intrinsic muscles. It is possible that these methods may not revert the deformity, but we are optimistic that it may increase the flexibility and strength of the muscles and the transverse arch’s function. This may prevent from developing deformities or to prevent the progression of a deformity. Future studies are needed to clarify the effects of these methods on the transverse arch, and at which intensity and duration it needs to be done to be effective. Furthermore, it is difficult to identify a one and only model for the perfect normal foot structure; therefore, it is important to assess the foot individually and determine the balance within it. To obtain a complete understanding of the transverse arch, it would be preferable to measure the structure and the function at the same time using devices such as the WPUID and individual force sensors. These methods help understand the mechanism of the structure and the loading transfers of the forefoot in hallux valgus with and without pain, and are also easy to use and affordable. The different results between studies so far may be caused by the type of sensors and machines used and the regions of force calculations, as well as whether participants are barefooted or have shoes on, the different adaptation methods to hallux valgus used to avoid pain by stepping on other parts of the foot, and the skin thickness and deformities in the lesser toes. To avoid further inconsistent results between studies about hallux valgus, it would be better to compare normal feet with hallux valgus feet with pain, or hallux valgus without pain, or hallux valgus with lesser toe deformities, each as a different group with different criteria. Understanding the detailed biomechanics of the foot is promising to improve foot health by pointing out which part to exercise and which part to reinforce with pads or orthoses [17, 18].

## Limitations

Our study has several limitations. Our sample size was small, and we used data from right and left feet, which may actually influence each other in walking and standing. We did not differentiate between the degrees of hallux valgus deformity and pain, and exact pain location. We took the measurements in barefoot condition and under static conditions, and the results could differ in individuals wearing shoes and under dynamic conditions. Future studies may consider these limitations to have better understanding of the forefoot structure.

## Conclusions

TAH and LS height seem to have an important role in assessing and choosing treatment protocols to hallux valgus deformity and forefoot pain. Our results contribute to the lacking research about the transverse arch of the foot and to the understanding of the structural and functional changes in hallux valgus with pain and without pain.

Further, the simple and low-cost methods that we have used do not exposure patients to harmful agents and may be helpful to therapists who are located away from big therapy centers and need to work without an interdisciplinary team.

## Abbreviations

NORM: normal feet group
HVG: feet with hallux valgus group
HV Pain (-): hallux valgus feet without pain group
HV Pain (+): hallux valgus feet with pain group
MS: medial sesamoid
LS: lateral sesamoid
1MTH: first metatarsal head
2MTH: second metatarsal head
3MTH: third metatarsal head
4MTH: fourth metatarsal head
5MTH: fifth metatarsal head
HVA: hallux valgus angle
TAH: transverse arch height
SRA: sesamoid rotation angle
pl: plantar surface
